# Classification of Crab-Field Rice and Conventional Rice Based on Multi-Element, Stable Isotope, and Non-Targeted Metabolome Combined with Chemometrics

**DOI:** 10.3390/foods14111853

**Published:** 2025-05-23

**Authors:** Xianxin Wu, Lina Li, Tianshu Peng, Qiujun Lin, Guang Li, Chunjing Guo, Xun Zou, Jianzhong Wang

**Affiliations:** Institute of Agricultural Quality Standards and Testing Technology, Liaoning Academy of Agricultural Sciences, Shenyang 110161, China; lln231118@163.com (L.L.); tspenglaas@163.com (T.P.); linqiujun85@163.com (Q.L.); lngtgh@163.com (G.L.); guocj464@163.com (C.G.); 18240260011@163.com (X.Z.)

**Keywords:** crab-field rice, multielements, stable isotopes, metabolomic, chemometrics, authenticity recognition

## Abstract

The quality of rice is closely related to its planting mode. The rice produced in rice–crab co-cultivation fields often enjoy higher prices and consumption enthusiasm than traditional rice due to the use of fewer chemical inputs, making it a key target of commercial fraud. In this study, multi-element, stable isotope, metabolite analysis techniques were synergistically applied with chemometric methods to distinguish between crab-field rice and common rice. Seven elements (Se, Rb, Cu, Cd, Ag, V, and Zn), two stable isotopes (*δ*^15^N and *δ*^13^C), and nine metabolites were identified as the most important discriminant variables. The discriminant analysis model based on seven elements and two stable isotopes, or based on nine metabolites, can completely distinguish between crab-field rice and conventional rice. The isotope, elemental, and metabolic fingerprint spectra selected in this study provide effective support for the authenticity identification of crab-field rice.

## 1. Introduction

Farmland and freshwater are important resources for ensuring a reliable global food supply. Maximizing productivity under limited water and land resources is crucial for sustainable agricultural development [1]. Rice (*Oryza sativa* L.) is a typical crop that relies on arable land and freshwater, feeding over 50% of the world’s population and an important component of global food security [2]. Therefore, determining how to obtain an efficient supply of rice while creating more added value under limited paddy field resources has become the mainstream development trend of agriculture today. It is gratifying that over 90% of the world’s rice paddies are in varying degrees of shallow water, providing favorable breeding conditions for aquatic animals such as fish, crabs, and shrimp [3], making it possible to develop a comprehensive agricultural aquaculture system, that is, to carry out aquaculture while cultivating rice. This ecological planting and breeding model of rice-aquaculture co-cultivation generates synergistic effects through the interaction of various components, which can achieve efficient internal circulation and system stability [4]. It has the advantages of improving freshwater and farmland utilization efficiency and system productivity, reducing the dependence on external energy and material inputs, and increasing economic benefits. In China, the integrated aquaculture area of rice and fishery and the production of aquatic products have exceeded 2 million hectares and 2.9 million tons, respectively [5]. The breeding targets include fish, shrimp, crab shells, amphibians, and reptiles, with a focus on the comprehensive cultivation of rice–shrimp and rice–fish in southern China, and rice–crab co-cultivation in northern China.

**Figure 1:**
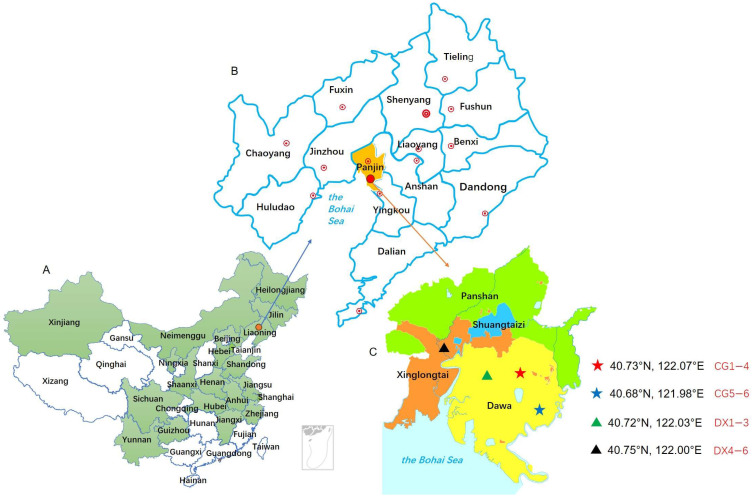
Geographic location information of sample source. (**A**) The provinces of China devoted to co-cultivation, (**B**) the enlargement of Liaoning province, and (**C**) the enlargement of Panjin city. CG represents rice obtained under conventional cultivation mode; DX represents rice obtained under rice–crab co-cultivation mode. In the map of China, the green-filled area represents provinces with rice lab integrated culture in China.

The Chinese mitten crab (*Eriocheir sinensis*) is often preferred as an aquatic species for co-cultivation with rice (*Oryza sativa* L.) in the ‘rice–crab co-cultivation’ ecological agriculture model [6], due to its fast growth rate, strong reproductive capacity, and high nutritional value [7]. Given the advantages of the dual harvesting of rice and crab, the comprehensive aquaculture area of rice and crab has increased sharply since the 1990s and has become a typical mutually beneficial and efficient ecological aquaculture model in many regions of China (Figure 1), especially in Liaoning Province [8,9]. Panjin City is located in the southern part of the Liao River Basin, serving as the estuary of the Liao River into the Bohai Sea [3]. It was the earliest region in Liaoning to develop the rice–crab co-culture model and is currently the best-developed area [2]. In the ecological planting method of rice–crab co-cultivation, crabs feed heavily on rice field pests, greatly reducing the use of fertilizers and pesticides in production. At the same time, in order to ensure the healthy growth of crabs, the application of highly toxic and residual pesticides is strictly restricted in rice production, making crab rice healthier and safer. On the other hand, the excrement of crabs can be transformed into natural organic fertilizers that are beneficial for rice growth through soil erosion and enhance the soil’s ability to retain water and nutrients, thereby improving the quality and nutritional value of rice. Given its superior safety and quality, the price of crab-field rice is usually higher than that of ordinary rice, enjoying strong market demand, high consumer recognition, and significant brand benefits.

Authenticity (authenticity of place of origin, authenticity of planting methods, authenticity of varieties, adulteration, etc.) has become the third major attribute of food or agricultural products after quality and safety, directly affecting consumers’ consumption enthusiasm, satisfaction, and happiness index. Especially in the context of the current shift in agricultural production from quantity meeting to quality improving, people’s psychological expectations for original, high-end, high nutritional value, and regional characteristic agricultural products are constantly increasing. As a result, these high-quality and distinctive agricultural products enjoy higher market prices and are more prone to fraudulent sales behaviors such as counterfeiting, substitution, and adulteration. Consumers will have to pay more for food with inaccurate labels [10]. Recently, ensuring the authenticity of food and agricultural products has become an important issue concerning the national economy and people’s livelihood, attracting high attention from governments, standard-setting organizations, and consumers [11]. The phenomenon of rice adulteration is common in the market, especially high-value rice, which is more susceptible to fraud, seriously affecting the production and sales of high-quality grains, consumer confidence, and food safety [12,13]. Therefore, rice quality and safety control, authenticity, and geographical source identification have become important research areas in food safety regulation [8]. Multi-element and stable isotope stoichiometry techniques have been considered advantageous and reliable methods for determining agricultural production methods and identifying geographical sources, due to their close relationship with the geographical environment, which have been widely used in the authenticity identification and origin tracing of agricultural products [14,15,16]. In addition, with the continuous development of high-throughput metabolomics technology, non-targeted metabolomics technology based on high-performance liquid chromatography–mass spectrometry has become an effective tool for analyzing the metabolic profiles of agricultural products, providing a powerful supplement to multi-element and isotope characterization techniques [17,18].

This article focuses on the conventional planting mode and rice–crab co-cultivation mode of rice in the typical rice ecological planting area (Panjin) in the Liaohe River Basin. The multi-element content, stable isotope ratio, and secondary metabolites of the two types of rice were comprehensively characterized, and the chemometric methods that can be used for identifying the authenticity of crab-field rice were explored. Furthermore, a data fusion strategy combining stable isotopes, multiple elements, and metabolites was adopted to further establish an accurate classification model. The results will establish an effective method to distinguish between crab-field rice and ordinary rice, ensuring the authenticity of crab-field rice and providing data support and theoretical supplements for the sustainable development of rice–crab co-cultivation ecological planting models.

## 2. Materials and Methods

### 2.1. Sample Collection and Preparation

The experiment mainly focused on Panjin rice, a well-known rice brand that is included in the China–Europe Mutual Recognition Geographical Indication Agricultural Products List. In this region, rice cultivation follows two distinct management modes: conventional planting and rice–crab co-cultivation. The primary difference between the two modes lies in crab feed management and nitrogen fertilizer application. In the rice–crab co-cultivation mode, crabs are provided with a formulated feed composed of soybean meal, wheat bran, cornmeal, and fish meal. However, the feed is placed in the field ridges adjacent to the rice paddies rather than directly applied into the fields, making it convenient for aquaculture personnel to check the feeding situation of river crabs and adjust the feeding amount in a timely manner. During the stage of supplementary fertilization, the rice–crab co-cultivation plots strictly prohibit the application of ammonium-based fertilizers (NH_4_^+^). Instead, urea is used as the sole nitrogen source, with a dosage capped at 5 kg per 667 m^2^. In terms of disease and pest control, the usage of chemical pesticides is less than that of conventional planting fields, and highly toxic pesticides are strictly used. Other than that, both conventional and rice–crab co-cultivation modes adopt identical protocols for application of base fertilizers, additional fertilizers other than nitrogen fertilizers, and other field management practices (e.g., soil type, irrigation, pest control). The samples were collected from the fields in the main planting area of Panjin during the rice maturity season. Composite samples were collected from each experimental plot using a five-point quincunx sampling method. A total of 60 rice subsamples were collected, including 30 from traditional planting fields and 30 from ecological planting fields where rice and crabs are co-cultivated. Five subsamples were systematically collected from the east, south, west, north, and center positions of each plot, then pooled to form a single representative composite sample per plot. This protocol ensured that spatial heterogeneity within individual fields was captured. A total of 12 experimental plots (including six both rice–crab co-cultivation and six conventional rice fields) yielded 12 composite samples after subsample integration. In order to eliminate the interference of varieties on the authenticity recognition of rice planting patterns, the most important cultivated variety in the Panjin area, Yanfeng 47, was selected for all rice samples. The specific information of the test sample groups is listed in Table 1. The geographical location information of the sample collection and its location in China are shown in Figure 1.

The rice ears of each sample were placed in large nylon mesh bags and air dried under direct sunlight. After threshing, shelling, and polishing the brown rice samples, each group of white rice samples was ground into a fine powder for multi-element and stable isotope analysis [14]. The fresh rice grain samples were processed according to the previous method to obtain polished rice [19], which was then frozen in liquid nitrogen and stored at −80 °C for subsequent metabolomic analysis.

### 2.2. Material Reagents and Instruments

All reagents and standards required for elemental analysis, isotope analysis, and metabolomics analysis are shown in Table 2, and information on specialized instruments and equipment is shown in Table 3. In order to avoid the cross-contamination of elements during the analysis process, the experimental procedure strictly followed the principle of separate storage of standards, strict separation of equipment storage rooms, and effective isolation of pre-processing.

### 2.3. Method for Multi-Element Analysis

About 0.5 g of milled rice powder (having passed 0.149 mm sieve) was weighed and placed into the 60 mL microwave oven digestion tank that had been pickled, 10.0 mL HNO_3_ was added, and then it was heated in the temperature control mode in the electrothermal digestion system (DigitBlock EHD36, LabTech) until the volume of digestive solution was reduced to about 0.5 mL. The cooled digestion solution was diluted to 25.0 mL with deionized water, mixed well, and filtered through a 0.45 μm aqueous membrane. ICP-MS (Agilent 7900, USA) and ICP-OES (Agilent 5800, USA) were used for analysis of 23 elements (Cu, Zn, K, P, Mg, Ca, Mn, Fe, As, Cd, Pb, Al, Rb, Se, Sr, V, Co, Ni, Ga, Cs, Ag, Cr, and Ba), in which ICP-MS was used to analyze Cu, Zn, As, Cd, Pb, Rb, Se, Sr, V, Co, Ni, Ga, Cs, Ag, and Cr elements, while ICP-OES was used to analyze K, P, Mg, Ca, Mn, Fe, Al, and Ba elements. The certified multi-element standard substance for powdered rice (GBW10043) was sourced from the National Research Center for Certified Reference Materials (Beijing, China).

### 2.4. Method for Stable Isotope Analysis

The stable isotope ratios of carbon, nitrogen, hydrogen, and oxygen in rice were measured by combining an elemental analyzer (Vario PYRO cube, Elementar, Germany) with an isotope ratio mass spectrometer (Isoprime 100, Elementar, Germany). About 5.0 mg of rice sample was weighed and put in a tin capsule (4 mm × 4 mm × 11 mm) for the measurement of C and N isotope ratios. The C and N elements in the rice sample were converted into pure CO_2_ and N_2_ through combustion, respectively, and detected using a stable isotope ratio mass spectrometer. The instrument parameters were set as follows: the combustion furnace temperature of the element analyzer is 920 °C, the reduction furnace temperature is 600 °C, the carrier gas was high-purity helium gas (He, 99.999%), the purge flow rate was 250 mL·min^−1^, the isotope ratio mass spectrometer detection time was 550 s, and the trap currents of CO_2_ and N_2_ were 100 μA and 400 μA, respectively.

When conducting the measurement of stable isotope ratios of hydrogen and oxygen in rice, about 1.0 mg of the rice sample was weighed in a silver capsule (4 mm × 4 mm × 11 mm) and placed in an automatic sampling tray of an element analyzer. After the H and O elements in the rice sample were thermally decomposed into H_2_ and CO, they were detected by a stable isotope ratio mass spectrometer. The instrument parameters were as follows: elemental analyzer, cracking furnace temperature of 1450 °C, He flow rate of 150 mL·min^−1^, isotope ratio mass spectrometry detection time of 950 s, and H_2_ and CO trap currents of 400 μA and 200 μA, respectively.

The calculation method for stable isotope ratios was to use a standard substance with known isotope ratios as a reference to calculate the relative value of stable isotope ratios for unknown samples. The specific calculation was disclosed as follows: *δ*X = (Rsample/Rstandard-1) × 1000‰ [14]. In the equation, Rsample and Rstandard represent the abundance ratios of heavy and light isotopes (^13^C/^12^C, ^15^N/^14^N, ^2^H/^1^H, and ^18^O/^16^O) in the measured sample and international standard sample, respectively. The test data were processed and calibrated using a two-point correction method, with *δ*^13^C and *δ*^15^N values corrected using USGS40, USGS90, and USGS91 standard material values. The reference materials for *δ*^2^H and *δ*^18^O were USGS55, USGS90, and USGS91 standard material values for calibration. Information on the specific reference materials is as follows: USGS90 (*δ*^2^H = −13.9‰, *δ*^18^O = +35.90‰, *δ*^13^C = −13.75‰, *δ*^15^N = +8.84‰); USGS91 (*δ*^2^H = −45.7‰, *δ*^18^O = +21.13‰, *δ*^13^C = −28.28‰, *δ*^15^N = +1.78‰); USGS55 (*δ*^2^H = −28.2‰, *δ*^18^O = +19.12‰); USGS40 (*δ*^13^C = −26.389‰, *δ*^15^N = −4.5‰).

### 2.5. Method for Metabolomics Analysis 

After removing the polished rice samples stored at −80 °C, they were frozen in liquid nitrogen and then ground using a mortar. About 100 mg of milled rice powder in each sample was put into a centrifuge tube, 500 μL of an 80% methanol aqueous solution was added and mixed evenly, and then the mixture was centrifuged at 1500 r/min for 20 min at 4 °C. The supernatant was diluted with water to a methanol content of 53% [20]. The 53% methanol aqueous solution was taken as the blank sample. Both the experimental samples and the blank sample were centrifuged at 1500 r/min for 20 min at 4 °C. The supernatant was collected and analyzed using a combination quadrupole Orbitrap liquid chromatography–mass spectrometer (Q Exactive™ HF-X, Thermo Fisher, MA, USA) [20]. The specific chromatographic and mass spectrometry conditions refer to previous methods [21]. The chromatographic column was determined to be a HypersilGold column (C18), with a column temperature of 40 °C and a flow rate of 0.2 mL/min. In positive mode, the 0.1% formic acid was used as mobile phase A, and methanol was used as the mobile phase B. In negative mode, 5 mM ammonium acetate (pH 9.0) was used as mobile phase A, and methanol was used as mobile phase B. The mass spectrometry scanning range was selected as *m*/*z* 100–1500. The settings of the ESI source were as follows: spray voltage of 3.5 kV, sheath gas flow rate of 35 psi, aux gas flow rate of 10 L/min, capillary temp of 320 °C, S-lens RF level of 60, and Aux gas heater temperature of 350 °C. Polarity was set to positive ion mode and negative ion mode, respectively. MS/MS secondary scanning was a type of data-dependent scan. The raw files obtained from mass spectrometry detection were imported into Compound Discoverer 3.3 software for spectral processing and a database search (MassList primary database and high-resolution secondary spectral databases mzCloud and mzVault) to obtain qualitative and quantitative results of metabolites. Then, background ions were removed from blank samples and standardized based on the formula ‘sample raw quantitative value/(total of sample metabolite quantitative values/total of QC1 sample metabolite quantitative values)’ to obtain the relative peak area. Finally, the identification and relative quantitative results of metabolites were obtained.

### 2.6. Statistical and Chemometric Analysis

One-way ANOVA was performed using Origin 2021 software to test the significance of differences among different rice groups. Each sample was analyzed in six replicates. Data were presented as the mean value ± standard deviation, with asterisks representing a significant level of difference between two sets of data (*p* < 0.05). Principal component analysis (PCA), orthogonal partial least-squares discriminant analysis (OPLS-DA), the variable importance projection score, box plots, and heatmaps were performed using MetaboAnalyst 4.0 online software (https://www.metaboanalyst.ca/), accessed on 20 December 2024.

## 3. Results and Discussion

### 3.1. Multielement Analysis

#### 3.1.1. Differential Analysis of Multi-Element Content Between Crab-Field Rice and Ordinary Rice

The multi-element content in crab-field rice and conventional rice was analyzed, and Table 4 shows the average values of 23 elements in rice under conventional planting mode and rice–crab co-cultivation mode. The contents of Cu, Cd, Rb, and Se in conventional rice were significantly higher than those in crab-field rice (*p* < 0.05); in contrast, the Ag content in crab-field rice is significantly higher than that in conventional rice. There was no significant difference in the content of other elements (Zn, K, P, Mg, Ca, Mn, Fe, As, Pb, Al, Sr, V, Co, Ni, Ga, Cs, Cr, and Ba) between crab-field rice and conventional rice. Comparative analysis revealed that Cu, Cd, Rb, Se, and Ag had potential contributions to distinguishing crab-field rice from conventional rice.

Among these elements, Ag, as a biophilic metal element, has bactericidal and disinfecting effects and is generally harmless to the human body. Studies have revealed that low-dose nano Ag can not only increase rice yield and tillering but also significantly enhance rice’s defense against abiotic (salt) and biotic (rice blast fungus and rice koji fungus) stresses, revealing the potential value of Ag in improving rice resistance [22,23]. In fact, our field research found that silver, as a particle-level and highly active silver element material, is an indispensable disinfectant material in crab farming due to its efficient antibacterial and bactericidal effects. Before being placed in rice paddies for cultivation, crab seedlings are likely to have ingested a certain amount of nanosilver. During their symbiosis with rice, they may release nanosilver into the rice paddies through a series of physiological activities. This may be the main reason why Ag in crab-field rice is significantly higher than that in conventional rice. Cd, classified as a Group 1 carcinogen by both the USA National Toxicology Program (NTP) and the International Agency for Research on Cancer (IARC), often causes dietary exposure through rice, promoting cancer progression through mechanisms including free-radical generation, the disruption of cellular proliferation and differentiation, oxidative DNA damage, and aberrant epigenetic modifications in tumor-associated gene promoter regions [24]. Previous studies on the enrichment and release characteristics of Cd by Chinese mitten crabs have shown that crabs have a strong ability to enrich heavy-metal Cd, with enrichment coefficients ranging from 6 to 3148. The biological half-life (B1/2) range of various tissues and organs is 8–57 days, and the overall release rate is relatively slow. Therefore, the decrease in Cd content in crab-field rice is closely related to the absorption of crabs [25]. As is also easily accumulated by rice. Our results indicated that the arsenic content in crab-field rice was slightly higher than that in regular rice, but the difference did not reach a significant level. Previous research has shown that the accumulation of As and Cd in rice grains under flooded conditions showed opposite trends [26], so the slight increase in As content in crab-field rice may be related to the significant decrease in Cd accumulation. Overall, the co-cultivation mode of rice and crab had a much greater effect on reducing heavy-metal Cd in rice than its promoting effect on As. Our results showed that the content of beneficial Ag and harmful Cd in crab-field rice was significantly higher and lower than that in conventional rice, respectively, revealing that the rice–crab co-cultivation model is more conducive to improving rice yield, resistance, and safety. This study provides further evidence of the ecological planting model of rice–crab co-cultivation to promote sustainable agricultural development. Focusing on Cu, in conventional rice production systems, Cu-containing Bordeaux mixture or copper-based fungicides are frequently applied to control plant diseases, leading to continuous Cu accumulation in soil. Rice plants absorb excess Cu through root uptake and subsequently transport it to grains for accumulation. In contrast, under rice–crab co-cultivation systems, the use of Cu-containing pesticides is strictly restricted to ensure crab health. In addition, crab activities such as soil turning and excrement decomposition can promote the formation of organic matter [27], which may fix Cu ions through chelation, reducing the mobility and plant effectiveness of Cu. Therefore, the content of Cu in our results is significantly lower in crab-field rice than in ordinary rice, which is in line with the production reality. Se is a component of glutathione peroxidase and plays a crucial role in aquaculture. Although crabs do not have a high demand for Se, it is indispensable. Therefore, the reduction in Se in crab-field rice may be related to the absorption of crabs. The decrease in Rb content in rice under the rice–crab co-culture mode may be related to the activity of crabs, but the specific mechanism still needs further research.

#### 3.1.2. Chemometric Analysis by Elemental Fingerprinting

**Figure 2:**
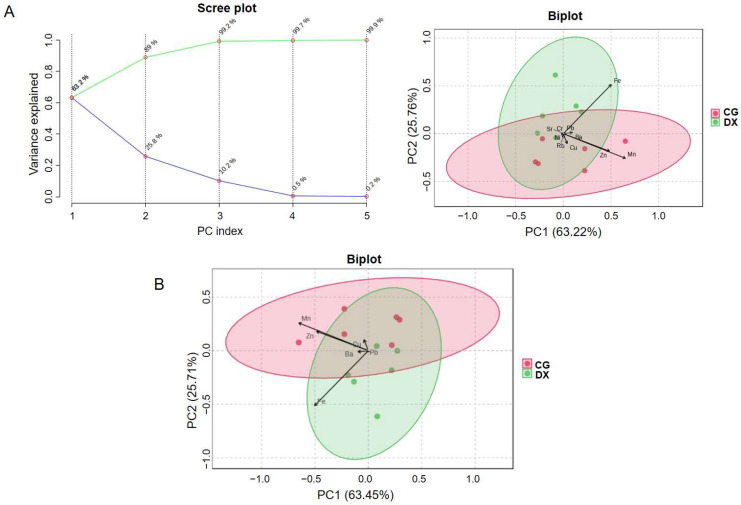
PCA model analysis based on multi-element fingerprint. (**A**) The scree plot and biplot of PCA based on 23 elements. In the scree plot, the lower line represents the variance percentage explained by each principal component and the upper line represents the cumulative variance percentage; (**B**) the biplot of PCA model based on the values of the selected elements (Mn, Fe, Zn, Ba, Cu, and Pb). CG represents rice obtained under conventional cultivation mode; DX represents rice obtained under rice–crab co-cultivation mode.

Due to the challenge of completely distinguishing similar groups using the significance level of a single variable between groups, introducing multivariate modeling methods to extract discriminative information from all variables will help to classify groups more clearly [28]. In order to explore the applicability of multiple elements in identifying the authenticity of rice produced in crab fields, an unsupervised modeling method (principal component analysis, PCA) was used first. As shown in the scree plot of PCA (Figure 2A), the cumulative contribution rate of principal component 1 and principal component 2 is over 80%, indicating that the PCA model established based on the content of these 23 elements has a good explanatory power on the original data. From the biplot of PCA (Figure 2A), it can be seen that the distance between the rice groups in the rice–crab co-cultivation field and those in the conventional planting field was relatively close, and there was some overlap of sample points within the confidence intervals of the two groups, indicating that the PCA model based on the 23 element fingerprints tested has poor performance in identifying the authenticity of crab-field rice. From the biplot in Figure 2A, it can also be seen that the six variables Mn, Fe, Zn, Ba, Cu, and Pb contribute the most to the principal components, while K, P, Mg, Ca, As, Cd, Al, Rb, Se, Sr, V, Co, Ni, Ga, Cs, Ag, and Cr had minimal contributions to the model. Therefore, we further constructed a PCA analysis model based on the values of these six selected elements, but it still cannot ideally distinguish rice samples between the rice–crab co-culture group and the conventional planting group (Figure 2B), with a discrimination accuracy of 60.0% for crab-field rice (the ratio of the correct number of samples interval to the total number of samples with 95% confidence).

**Figure 3:**
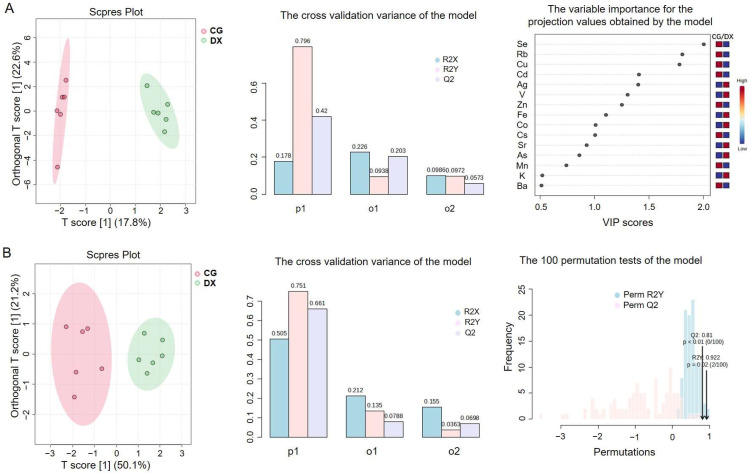
OPLS-DA model analysis based on multi-element fingerprint. (**A**) The OPLS-DA model based on fingerprint 23 elements; (**B**) the OPLS-DA model based on the 7 feature parameters (Se, Rb, Cu, Cd, Ag, V, and Zn). In the cross-validation variance of the model, P1 represents the variance contribution of the prediction set, reflecting the predictive ability of the model on the prediction set data, that is, the performance of the model on unknown data; O1 represents the variance contribution of the orthogonal prediction set; O2 represents the variance contribution of the orthogonal residual set; R2X indicates the cumulative variance explained by the model for the independent variable X; R2Y indicates the cumulative variance explained by the model for the dependent variable Y (grouping). Q2 reflects the predictive power of the model, i.e., the model’s ability to predict the variance of the data. CG represents rice obtained under conventional cultivation mode; DX represents rice obtained under rice–crab co-cultivation mode.

Compared to PCA, partial least-squares discriminant analysis (PLS-DA) is a supervised chemometric method that maximizes the separation between different groups, thereby achieving better classification and prediction [29]. Orthogonal partial least-squares discriminant analysis (OPLS-DA) decomposes the X matrix information into Y-related and unrelated parts through orthogonal signal correction and PLS-DA, which can more effectively filter out variables unrelated to classification, maximize group differentiation, and help discover differential biomarkers [30]. To solve the problem of poor performance of the PCA method in intergroup classification, the supervised chemometric model (OPLS-DA), commonly used to distinguish between two sets of data, was employed. Figure 3A shows the OPLS-DA model based on multi-element fingerprint, indicating that OPLS-DA has certain advantages in distinguishing crab-field rice samples from ordinary rice samples compared with PCA. The cross-validation variance of the OPLS-DA model shows that R^2^ = 0.796, indicating that the model’s explanatory power of the data is acceptable, while Q^2^ < 0.5 indicates that the model’s predictive ability was poor. In the variable importance for the projection (VIP) values obtained by the OPLS-DA model, the parameters with VIP > 1.0, including Se, Rb, Cu, Cd, Ag, V, Zn, Fe, Co, and Cs, are considered to have significant contributions in model interpretation. Multiple rounds of variable combination modeling analyses revealed that models incorporating Se, Rb, Cu, Cd, Ag, V, and Zn had the best discrimination effect between groups, significantly better than the model with Fe, Co, or Cs included. Therefore, an OPLS-DA model based on these seven feature parameters was further constructed to better achieve authenticity recognition of crab-field rice. As shown in Figure 3B, the model has a good discrimination effect on rice from rice–crab co-cultivation fields and conventional planting fields. However, the corresponding cross-validation test plot with R^2^ = 0.751 and Q^2^ = 0.661 indicates that the model has relatively acceptable explanatory power for the data and predictive ability. According to the results of 100 permutation tests of the model, the *p*-value of Q^2^ is less than 0.01, indicating that the current model is already optimal in terms of predictive ability. The *p*-value of R^2^ is 0.02, indicating that from the perspective of the model’s data interpretation rate, two random grouping models will be better than the current model, proving that the OPLS-DA model constructed based on seven elements fits well but did not reach the optimal level for the accurate discrimination of samples.

Based on OPLS-DA screening, it was found that Cu, Cd, Rb, Se, and Ag overlap with the five elements revealed in Section 3.1.1, which have significant differences between crab-field rice and ordinary rice groups. In addition, two other elements involved are Zn and V, with Zn having a higher content in regular rice and V having a higher content in crab-field rice. Zn is an essential element for crab growth, so the decrease in Zn content in crab rice may be related to crab uptake. The activity of crabs often promotes the decomposition of organic matter and nutrient mineralization. Trace elements, including vanadium, may be released from sediments through microbial action and transformed into forms that can be absorbed by rice, resulting in an increase in V content in crab-field rice.

### 3.2. Stable Isotope Analysis

#### 3.2.1. Differential Analysis of Stable Isotopes Between Crab-Field Rice and Ordinary Rice

**Figure 4:**
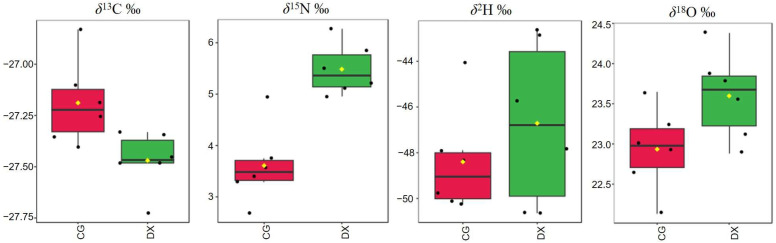
The box plot for isotopic ratio distribution trend of all rice samples. CG represents rice obtained under conventional cultivation mode; DX represents rice obtained under rice–crab co-cultivation mode. Six samples were measured for each group, and the data for each sample were repeated three times.

Stable isotope values in rice under different cultivation modes were detected to explore characteristic markers that can be used for authenticity identification of crab-field rice. The mean values of stable isotopes (*δ*^13^C, *δ*^15^N, *δ*^2^H, and *δ*^18^O) in crab-field rice and conventional rice are listed in Table 5. Statistical analysis revealed significant differences in the values of *δ*^13^C and *δ*^15^N between crab-field rice and conventional rice, while there was no significant difference in the values of *δ*^2^H and *δ*^18^O between the two groups of rice. The isotopic ratio distribution trend of all rice samples can be seen in the box plot (Figure 4). In conventional rice, the values of *δ*^13^C, *δ*^15^N, *δ*^2^H, and *δ*^18^O range from −27.40‰ to −26.83‰, 2.69‰ to 4.95‰, −50.25‰ to −44.07‰, and 22.13‰ to 23.64‰, respectively. The range of variation for the values of δ^13^C, *δ*^15^N, *δ*^2^H, and *δ*^18^O in crab rice is −27.73~−27.33‰, 4.95~6.27‰, −50.60~−42.85‰, and 23.11~24.38‰, respectively. Comparing and analyzing the stable isotope ratio distribution trends between crab-field rice and conventional rice, the threshold ranges of *δ*^15^N and *δ*^13^C in crab-field rice are significantly higher and lower than those in conventional rice, respectively. However, there is a significant overlap in the box distribution areas of *δ*^2^H and *δ*^18^O in the two groups of rice. The above results indicate that *δ*^15^N and *δ*^13^C can be used as identification parameters for crab-field rice.

*δ*^15^N has been proven by previous studies to be an effective parameter for distinguishing organic and traditional agricultural systems, as organic fertilizers are typically enriched in ^15^N compared to synthetic fertilizers, due to isotope fractionation caused by NH_3_ volatilization during storage or processing of organic fertilizers [31]. Moreover, the influence of fertilizer type on the *δ*^15^N value is significantly higher than that of regional differences [32]. There are often significant differences in the fertilizers enjoyed by crab-field rice and conventional rice during the planting process, especially during the stage of additional fertilization, whereby the type of nitrogen fertilizer used in rice–crab co-cultivation fields must be organic and the amount used must be significantly lower than that in conventional rice fields to ensure the healthy growth of crabs. Previous studies have shown that compared to conventional planting methods, the reduction rate of nitrogen fertilizer input in rice–crab co-cultivation fields is 20–30% [27]. Our results showed that *δ*^15^N in crab-field rice grown in organic ecology was significantly higher than that in conventional rice, which is consistent with previous research findings [14,31]. Recent studies have shown that under the high-density crab–rice co-cultivation mode, CH_4_ emissions were significantly reduced by more than 40% compared to conventional rice cultivation [27], which partially explains why the *δ*^13^C value in rice grown under the ecological planting model of rice–crab co-cultivation is lower than that of rice grown under the conventional model in this study. In summary, our results further revealed that the rice–crab co-cultivation model was one of the important strategies for reducing nitrogen and carbon in sustainable agricultural development.

*δ*^2^H and *δ*^18^O are closely related to geographical environmental factors such as temperature, latitude, altitude, precipitation, climate, irrigation water, and evaporation, and are therefore often used as landmark indicators reflecting the origin of agricultural products [33,34]. In this study, the focus is on the impact of planting patterns on rice, and there is no significant geographical isolation in the sample collection area. Our results revealed that there is no significant difference in *δ*^2^H and *δ*^18^O values between crab-field rice and conventional rice is reasonable.

#### 3.2.2. Chemometric Analysis by Stable Isotope Fingerprinting

**Figure 5:**
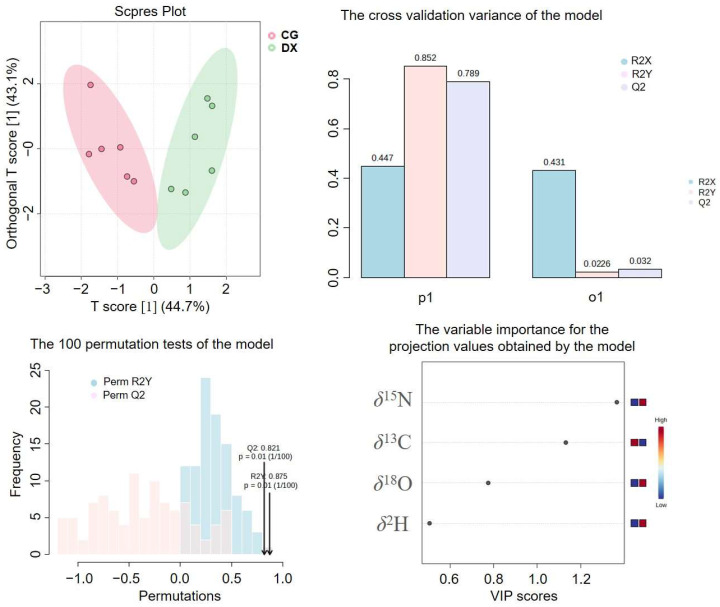
OPLS-DA model analysis based on stable isotopes. P1 represents the variance contribution of the prediction set, reflecting the predictive ability of the model on the prediction set data, that is, the performance of the model on unknown data; O1 represents the variance contribution of the orthogonal prediction set; R2X indicates the cumulative variance explained by the model for the independent variable X; R2Y indicates the cumulative variance explained by the model for the dependent variable Y (grouping); Q2 reflects the predictive power of the model, i.e., the model’s ability to predict the variance of the data. CG represents rice obtained under conventional cultivation mode; DX represents rice obtained under rice–crab co-cultivation mode.

Due to the limited number of parameters used for stable isotope analysis, PCA analysis was not suitable. The applicability of stable isotope fingerprinting in identifying the authenticity of crab-field rice was analyzed using OPLS-DA chemometrics. The OPLS-DA model established based on the four stable isotopes (*δ*^13^C, *δ*^15^N, *δ*^2^H, and *δ*^18^O) is shown in Figure 5. It can be seen that the crab-field rice group and the conventional rice group have been effectively distinguished. From the cross-validation variance of the OPLS-DA model, the R^2^ and Q^2^ were 0.852 and 0.789, indicating that the model has good explanatory power for the data and predictive ability. The permutation testing results showed that the *p*-values of R^2^Y and Q^2^ are both 0.01, indicating that the current model fits well, but in 100 permutation tests, there will be one random model that outperforms the current model. The VIP values reflect the contribution rates of different parameters to the grouping, in which *δ*^15^N and *δ*^13^C with VIP greater than 1 are potential feature identification indicators for distinguishing crab-field rice from conventional rice. This conclusion is consistent with the results of the box plots and average values.

### 3.3. Joint Analysis of Multiple Elements and Stable Isotopes

**Figure 6:**
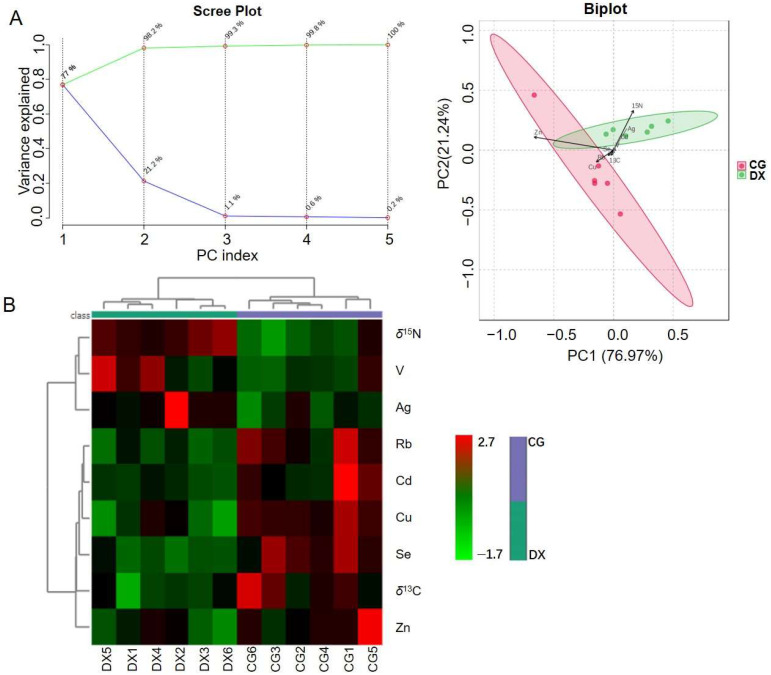
Unsupervised analysis based on selected characteristic elements (Se, Rb, Cu, Cd, Ag, V, and Zn) and stable isotopes (*δ*^15^N and *δ*^13^C). (**A**) TPCA model: in the scree plot, the lower line represents the variance percentage explained by each principal component and the upper line represents the cumulative variance percentage; (**B**) heatmap of hierarchical cluster analysis: CG represents rice obtained under conventional cultivation mode; DX represents rice obtained under rice–crab co-cultivation mode.

Recently, stable isotope and multi-element analysis techniques have been commonly combined for geographical source identification of agricultural products and have shown better discrimination accuracy than relying on a single analysis technique [15,18]. Considering that the authenticity recognition models for crab-field rice based solely on multi-element or stable isotopes need to be optimized, we combined the selected characteristic elements (Se, Rb, Cu, Cd, Ag, V, and Zn) and stable isotopes (*δ*^15^N and *δ*^13^C) that contribute significantly to distinguishing crab-field rice from conventional rice, and further established the model through unsupervised and supervised chemometric methods. The model established based on unsupervised PCA chemometrics shows that the cumulative contribution rate of the two principal components is as high as 98.2% and can achieve 100% differentiation between the tested crab-field rice and conventional rice (Figure 6A). It can be seen that, both in terms of the ability to interpret data and the accuracy of discriminant analysis, the model established by combining multiple elements and stable isotopes is far superior to the model established solely based on multiple elements or stable isotopes. From the heat map established by another unsupervised chemometric method (hierarchical cluster analysis), it can be found that based on the nine selected characteristic elements and stable isotope fingerprint information, crab-field rice samples and conventional rice samples were automatically divided into two categories (Figure 6B). Compared with conventional rice, crab-field rice has higher values of *δ*^15^N(‰), Ag, and V, while the values of Rb, Cd, Cu, Se, *δ*^13^C(‰), and Zn in crab-field rice are relatively low.

**Figure 7:**
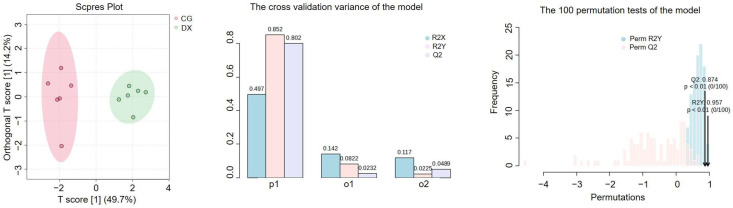
The supervised OPLS-DA model analysis based on selected characteristic elements (Se, Rb, Cu, Cd, Ag, V, and Zn) and stable isotopes (*δ*^15^N and *δ*^13^C). In the cross-validation variance of the model, P1 represents the variance contribution of the prediction set, reflecting the predictive ability of the model on the prediction set data, that is, the performance of the model on unknown data; O1 represents the variance contribution of the orthogonal prediction set; O2 represents the variance contribution of the orthogonal residual set; R2X indicates the cumulative variance explained by the model for the independent variable X; R2Y indicates the cumulative variance explained by the model for the dependent variable Y (grouping); Q2 reflects the predictive power of the model, i.e., the model’s ability to predict the variance of the data. CG represents rice obtained under conventional cultivation mode; DX represents rice obtained under rice–crab co-cultivation mode.

The supervised OPLS-DA method was further implemented to establish the classification model (Figure 7). The OPLS-DA model established based on the nine selected characteristic parameters can effectively distinguish crab-field rice from conventional rice. The cross-validation variance plot shows that the R^2^Y value reflecting the model’s ability to interpret data is 0.852, which is higher than the R^2^Y value of the OPLS-DA model based solely on multi-element fingerprints, and is on par with the R^2^Y value of the model based solely on stable isotope fingerprints. The Q^2^ value reflecting the predictive ability of the model is 0.802, which is higher than Q^2^ for models based solely on multiple elements or stable isotopes. The results of 100 permutation tests show that the corresponding *p*-values for R^2^Y and Q^2^ are both less than 0.01, indicating that the current model is already optimal. In summary, Se, Rb, Cu, Cd, Ag, V, Zn, *δ*^15^N, and *δ*^13^C can be combined for the authenticity recognition of crab-field rice, with a recognition accuracy of up to 100%. Moreover, whether using unsupervised or supervised chemometric analysis methods, the discriminant analysis performance of models established by combining multiple elements and stable isotopes is better than models established solely based on elements or stable isotopes. This result demonstrates the application prospects and advantages of combining multiple analysis techniques based on different parameters in the identification of agricultural product authenticity. However, determining how to achieve the maximum integration of various identification and analysis techniques without increasing time and economic costs remains a key issue to be solved in the future.

### 3.4. Metabolomic Analysis

The composition of secondary metabolites during plant growth is the result of the combined effects of various factors such as variety, the external climate environment (temperature, humidity, precipitation, soil, etc.), and cultivation methods [35]. Metabolomics, as an important component of systems biology, is capable of comprehensive and unbiased analysis of a large number of small-molecule metabolites in agricultural products, having demonstrated unique advantages in the field of authenticity identification of agricultural products in recent years [36]. In this study, based on untargeted metabolomics analysis, a total of 869 metabolites were screened in positive ion mode (Table S1) and 706 metabolites were screened in negative ion mode (Table S2) in all rice samples tested. VIP analysis was conducted on the contribution of these metabolites in distinguishing crab-field rice from conventional rice using the OPLS-DA method. Usually, variables with VIP > 1.0 are considered key components in the classification, and variables with VIP > 2.0 are identified as the most significant discriminant factors [37]. Due to the large number of metabolites with VIP > 1.0, we have raised the judgment criteria to VIP > 2.0 to obtain the most significant factors for discrimination. In positive ion mode, the contribution rate VIP value of 23 metabolites to distinguish between the conventional control group and the rice–crab co-culture group is greater than 2.0 (Figure S1). Under negative ion mode, the contribution rate VIP value of 19 metabolites to distinguish between the conventional control group and the rice–crab co-culture group is greater than 2.0 (Figure S2).

Further analysis was conducted on the nutrition and function of these metabolites, as well as their potential existence in rice, through literature and data searches. The results showed that among the metabolites with a contribution rate VIP greater than 2.0 used to distinguish inter group differences in positive ion mode, 10 were reported to exist in rice, including LPC (1-acyl 16:0), LPC (1-acyl 18:2), L-Glutathione oxidized, LPC 15:1-SN1, LPC 14:0-SN1, LPC 18:1-SN1, LPC 16:2-SN1, Folinic acid, LPC 16:0-SN1, and LPC 18:2-SN1. In negative ion mode, eight metabolic species with VIP contributions greater than 2.0 were reported to be likely to be real metabolites in rice, namely LysoPC 15:1, Guanosine, D-Sphingosine, D-(+)-Tryptophan, Acetylcysteine, Lysope 16:0, GMP, and Cyclic ADP-ribose. Based on the relative content fingerprints of these 18 metabolites, an OPLS-DA model was constructed (Figure S3). In the cross-validation variance plot, R^2^Y and Q^2^ are both greater than 0.8, indicating that the model has good explanatory power for the data and predictive ability (Figure S3). However, the results of 100 permutation tests show that the *p*-value is not less than 0.01, indicating that there is still room for optimization in the current model (Figure S3).

**Figure 8:**
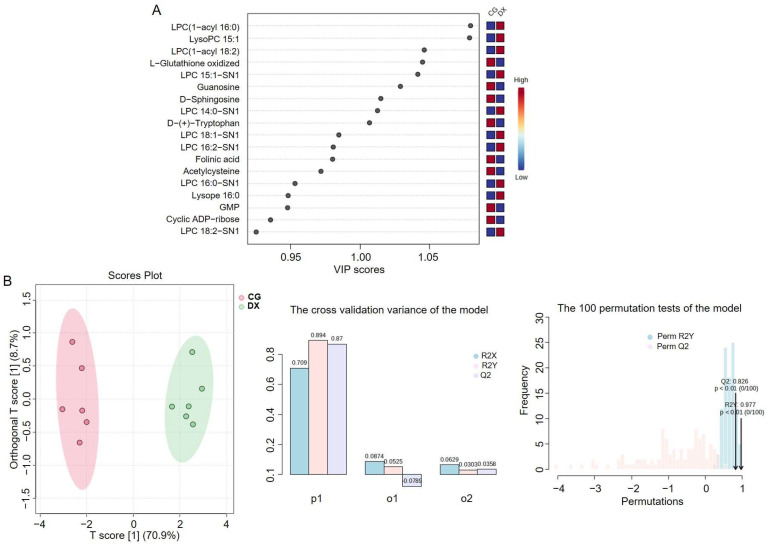
The optimized OPLS-DA model analysis based on selected characteristic metabolites with VIP > 1.0. (**A**) Screening of grouping indicative metabolites with VIP > 1 from 18 metabolites; (**B**) the OPLS-DA model based on nine characteristic metabolites selected from (**A**). In the cross-validation variance of the model, P1 represents the variance contribution of the prediction set, reflecting the predictive ability of the model on the prediction set data, that is, the performance of the model on unknown data; O1 represents the variance contribution of the orthogonal prediction set; O2 represents the variance contribution of the orthogonal residual set; R2X indicates the cumulative variance explained by the model for the independent variable X; R2Y indicates the cumulative variance explained by the model for the dependent variable Y (grouping); Q2 reflects the predictive power of the model, i.e., the model’s ability to predict the variance of the data. CG represents rice obtained under conventional cultivation mode; DX represents rice obtained under rice–crab co-cultivation mode.

Therefore, we further selected metabolites with VIP > 1.0 (five metabolites under positive ion mode: LPC(1-acyl 16:0), LPC(1-acyl 18:2), L-Glutathione oxidized, LPC 15:1-SN1, and LPC 14:0-SN1; four metabolites under negative ion mode: LysoPC 15:1, Guanosine, D-Sphingosine, and D-(+)-Tryptophan) among these 18 variables to construct a better model (Figure 8A). As shown in Figure 8B, the newly optimized OPLS-DA model can completely distinguish the two groups, with R^2^Y and Q^2^ both close to 0.9, indicating that the model has excellent data interpretation and prediction ability. The results of 100 permutation tests show that the *p*-value are both less than 0.01, indicating that the current model is already optimal.

**Figure 9:**
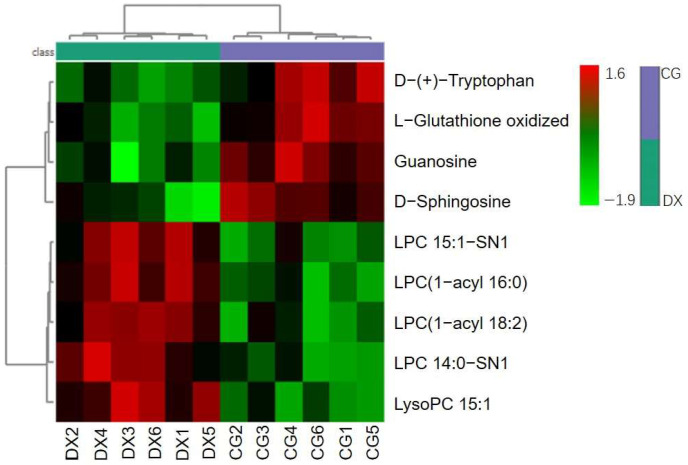
The heatmap of hierarchical cluster analysis based on nine characteristic metabolites. CG represents rice obtained under conventional cultivation mode; DX represents rice obtained under rice–crab co-cultivation mode.

**Figure 10:**
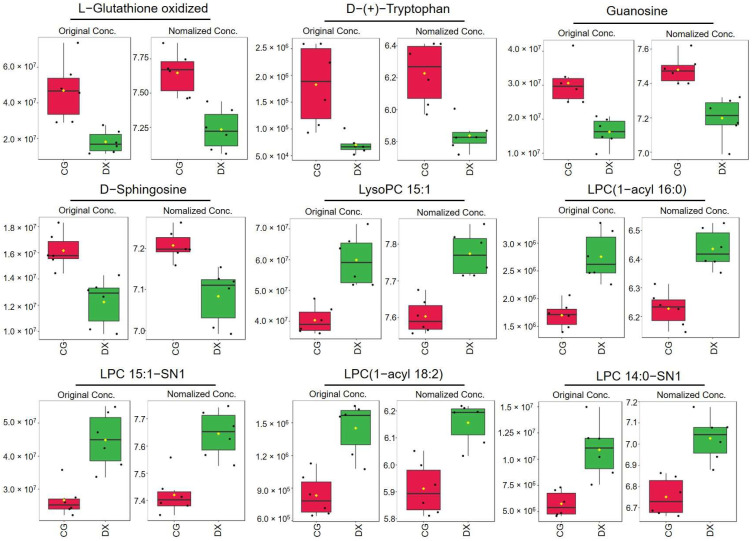
The box plot for the nine characteristic metabolites’ distribution trend of all rice samples. The left graph corresponding to each metabolite is based on the original relative content value, and the right graph is based on the logarithmic standardized value. CG represents rice obtained under conventional cultivation mode; DX represents rice obtained under rice–crab co-cultivation mode.

In addition, we conducted hierarchical clustering analysis based on the selected characteristic metabolites used to distinguish between crab-field rice and conventional rice. Figure 9 shows the heatmap obtained based on nine characteristic metabolites, indicating that crab-field rice and conventional rice are effectively classified into two categories. This result further confirms the potential value of these nine metabolites in identifying the authenticity of crab-field rice. We also constructed box plots to further illustrate the relative distribution trends of these nine metabolites in crab-field rice and conventional rice (Figure 10). The results showed that the relative contents of L-Glutathione oxidized, D-(+)-Tryptophan, Guanosine, and D-Sphingosine in conventional rice were higher than those in crab-field rice, while the relative contents of LysoPC 15:1, LPC (1-acyl 16:0), LPC 15:1-SN1, LPC (1-acyl 18:2), and LPC 14:0-SN1 in crab-field rice were significantly higher than those in conventional rice. Overall, the distribution trends of the nine metabolites in crab-field rice and conventional rice reflected by OPLS-DA, boxplots, and heatmaps are consistent. It is worth noting that the characteristic markers with significantly higher content in crab-field rice are all lysophosphatidylcholine (LPC) metabolites, which may be related to the increase in LPC synthesis precursors in soil that can be absorbed by rice plants through crab excrement. LPC is a type of lysophospholipid that has been shown to make significant contributions in indicating the origin and storage time of rice [38,39]. Our research provides new ideas for the application of LPC in identifying the authenticity of planting methods for agricultural products. In addition, the lipid content in rice is also a major factor determining the taste and appearance of rice. Studies have shown that LPC often forms a complex with linear starch, which may reduce the swelling power, solubility, and starch digestibility of rice, making it less likely for rice to gelatinize and resulting in more glossy and delicious rice after cooking [40]. This study revealed that the relative content of LPC in crab-field rice is higher than that in ordinary rice, further enriching the advantages of crab-field rice in terms of taste quality and providing data support for the high price of crab-field rice.

## 4. Conclusions

This study preliminarily explored the potential applications of multi-element, stable isotope, and metabolite identification in the authenticity of crab-field rice. The results indicate that rice produced under the rice–crab co-cultivation ecological planting mode and the traditional planting mode has different element contents and isotope ratios. Among these variables, Se, Rb, Cu, Cd, Ag, V, Zn, *δ*^15^N, and *δ*^13^C have a high correlation with planting patterns. There is room for optimization in discriminant analysis models that rely solely on characteristic elements or stable isotopes. The unsupervised PCA model, HCA heatmap, and supervised OPLS-DA model established by combining characteristic elements (Se, Rb, Cu, Cd, Ag, V, and Zn) and stable isotopes (*δ*^15^N and *δ*^13^C) can completely distinguish tested crab-field rice from conventional rice with a discrimination accuracy of 100%. Based on non-targeted metabolomics combined with OPLS-DA, boxplots, and HCA analysis, nine characteristic metabolites were screened that can effectively distinguish crab-field rice from conventional rice, namely, L-Glutathione oxidized, D-(+)-Tryptophan, Guanosine, D-Sphingosine, LysoPC 15:1, LPC (1-acyl 16:0), LPC 15:1-SN1, LPC (1-acyl 18:2), and LPC 14:0-SN1. The results of this study provide important data and technical support for the identification and brand protection of high-priced crab rice and have certain significance for maintaining market fairness and protecting the rights and interests of producers and consumers. However, considering that the model established in this study is based on a small and geographically limited sample set and may not be applicable to a wider range of scenarios, it is necessary to expand the sample size, enrich the sample collection period and sources, and optimize the discriminant analysis model in the future to effectively increase the scientificity, convenience, and universality of the crab-field rice authenticity recognition method.

## Figures and Tables

**Table 1:** Specific information about the test sample.

Sample Group Number	Sampling Sites	Planting Patterns	Rice Variety
CG1	Liujia Village, Tangjia Town, Dawa District, Panjin City	Traditional planting mode	Yanfeng 47
CG2	Liujia Village, Tangjia Town, Dawa District, Panjin City	Traditional planting mode	Yanfeng 47
CG3	Liujia Village, Tangjia Town, Dawa District, Panjin City	Traditional planting mode	Yanfeng 47
CG4	Liujia Village, Tangjia Town, Dawa District, Panjin City	Traditional planting mode	Yanfeng 47
CG5	Bianwazi Village, Xi’an Town, Dawa District, Panjin City	Traditional planting mode	Yanfeng 47
CG6	Bianwazi Village, Xi’an Town, Dawa District, Panjin City	Traditional planting mode	Yanfeng 47
DX1	Sanjiazi Village, Chengjiao Township, Dawa Street, Panjin City	Ecological planting mode of rice–crab co-cultivation	Yanfeng 47
DX2	Sanjiazi Village, Chengjiao Township, Dawa Street, Panjin City	Ecological planting mode of rice–crab co-cultivation	Yanfeng 47
DX3	Sanjiazi Village, Chengjiao Township, Dawa Street, Panjin City	Ecological planting mode of rice–crab co-cultivation	Yanfeng 47
DX4	Xinsheng Street, Xinglongtai District, Panjin City	Ecological planting mode of rice–crab co-cultivation	Yanfeng 47
DX5	Xinsheng Street, Xinglongtai District, Panjin City	Ecological planting mode of rice–crab co-cultivation	Yanfeng 47
DX6	Xinsheng Street, Xinglongtai District, Panjin City	Ecological planting mode of rice–crab co-cultivation	Yanfeng 47

Note: CG represents rice obtained under conventional cultivation mode; DX represents rice obtained under rice–crab co-cultivation mode.

**Table 2:** Specific information about the test reagents.

Reagents	Purity	Brand	Origin
Methanol	LC-MS Grade	Thermo Fisher, Waltham, MA, USA	USA
H_2_O	LC-MS Grade	Merck, Darmstadt, Germany	Germany
Formic acid	LC-MS Grade	Thermo Fisher	USA
Ammonium acetate	LC-MS Grade	Thermo Fisher	USA
Nitric acid	Guaranteed reagent	Merck	Germany
Standard for multi-element analysis (GBW10043)	—	National Research Center for Certified Reference Materials	China
Standards for multi-element analysis (USGS40, USGS90, USGS91,USGS55)	—	United States Geological Survey	USA

**Table 3:** Specific information about the instruments and equipment.

Name	Model	Brand	Origin
ICP-OES	5800	Agilent, Santa Clara, CA, USA	USA
ICP-MS	7900	Agilent	USA
Graphite digestion instrument	ST36-iTOUCH	LabTech, Cambridge, MA, USA	USA
Elemental analyzer	Vario PYRO cube	Elementar, Langenselbold, Germany	Germany
Isotope ratio mass spectrometer	Isoprime 100	Elementar	Germany
Low-temperature centrifuge	D3024R	Scilogex, Tarrytown, NY, USA	USA
Orbitrap liquid chromatography-mass spectrometer	Q Exactive™ HF/Q Exactive™ HF-X	Thermo Fisher	USA
Chromatographic column	Hypesil Gold column C18 (100 × 2.1 mm, 1.9 μm)	Thermo Fisher	USA

**Table 4:** The mean values of the 23 elements in crab-field rice and conventional rice.

Element/Unit	CG	DX
Cu (mg/kg) *	2.274 ± 0.300	1.427 ± 0.472
Zn (mg/kg)	13.368 ± 2.160	11.155 ± 1.385
K (g/100 g)	0.099 ± 0.025	0.107 ± 0.014
P (mg/kg)	0.112 ± 0.025	0.112 ± 0.012
Mg (g/100 g)	0.032 ± 0.007	0.033 ± 0.005
Ca (g/100 g)	0.008 ± 0.002	0.007 ± 0.001
Mn (mg/kg)	10.207 ± 3.375	8.528 ± 1.303
Fe (g/100 g)	4.462 ± 2.662	6.450 ± 2.148
As (mg/kg)	0.167 ± 0.034	0.185 ± 0.020
Cd (mg/kg) *	0.004 ± 0.003	0.001 ± 0.001
Pb (mg/kg)	0.295 ± 0.181	0.253 ± 0.156
Al (g/100 g)	0.003 ± 0.002	0.003 ± 0.001
Rb (mg/kg) *	0.617 ± 0.225	0.264 ± 0.094
Se (mg/kg) *	0.031 ± 0.009	0.012 ± 0.004
Sr (mg/kg)	0.269 ± 0.065	0.313 ± 0.038
V (mg/kg)	0.002 ± 0.001	0.003 ± 0.002
Co (mg/kg)	0.003 ± 0.002	0.010 ± 0.013
Ni (mg/kg)	0.054 ± 0.015	0.053 ± 0.029
Ga (mg/kg)	0.001 ± 0.001	0.001 ± 0.001
Cs (mg/kg)	0.002 ± 0.001	0.002 ± 0.001
Ag (mg/kg) *	0.013 ± 0.005	0.022 ± 0.006
Cr (mg/kg)	0.044 ± 0.024	0.055 ± 0.040
Ba (mg/kg)	0.411 ± 0.558	0.212 ± 0.206

Note: CG represents rice obtained under conventional cultivation mode; DX represents rice obtained under rice–crab co-cultivation mode. Each sample was analyzed in six replicates. Data are represented as the mean ± SD. Asterisks (*) indicate statistically significant differences between groups (*p* < 0.05).

**Table 5:** The mean values of stable isotopes in crab-field rice and conventional rice.

Mode	*δ*^13^C (‰)	*δ*^15^N (‰)	*δ*^2^H (‰)	*δ*^18^O (‰)
CG	−27.188 ± 0.207	3.609 ± 0.749	−48.397 ± 2.332	22.934 ± 0.519
DX	−27.469 ± 0.142 *	5.485 ± 0.500 *	−46.712 ± 3.586	23.598 ± 0.542

Note: CG represents rice obtained under conventional cultivation mode; DX represents rice obtained under rice–crab co-cultivation mode. Data are represented as the mean ± SD (*n* = 6). Asterisks (*) indicate statistically significant differences between groups (*p* < 0.05).

## Data Availability

Data are contained within the article.

## Supplementary Materials

The following supporting information can be downloaded at: https://www.mdpi.com/article/10.3390/foods14111853/s1, Figure S1: VIP plot for contribution of parameters distinguishing crab field rice from conventional rice in positive ion mode; Figure S2: VIP plot for contribution of parameters distinguishing crab field rice from conventional rice in negative ion mode; Figure S3: OPLS-DA model analysis based on 18 metabolites. (A): the OPLS-DA model based on multi-element fingerprint; (B): the cross validation variance of (A); (C): shows the results of 100 permutation tests of (A); CG represents rice obtained under conventional cultivation mode; DX represents rice obtained under rice crab co cultivation mode; Table S1: All metabolites and their relative contents in the tested rice samples screened under positive ion mode; Table S2: All metabolites and their relative contents in the tested rice samples screened under negative ion mode.
