# Supplementary material for: Classification of Crab-Field Rice and Conventional Rice Based on Multi-Element, Stable Isotope, and Non-Targeted Metabolome Combined with Chemometrics
Source: Foods. 2025 May 23;14(11):1853. doi: 10.3390/foods14111853 (PMC12154286; doi:10.3390/foods14111853)
Supplement: Supplementary file 1 [file foods-14-01853-s001.zip › foods-3614560 Supplemental figures.pdf]

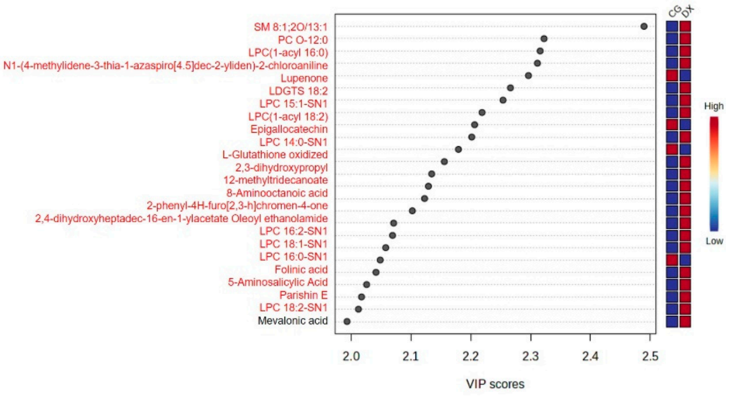


**Figure S1** VIP plot for contribution of parameters distinguishing crab field rice from conventional rice in positive ion mode

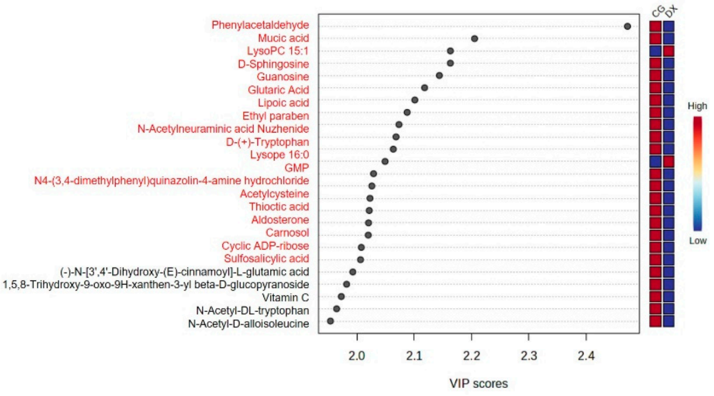


**Figure S2** VIP plot for contribution of parameters distinguishing crab field rice from conventional rice in negative ion mode

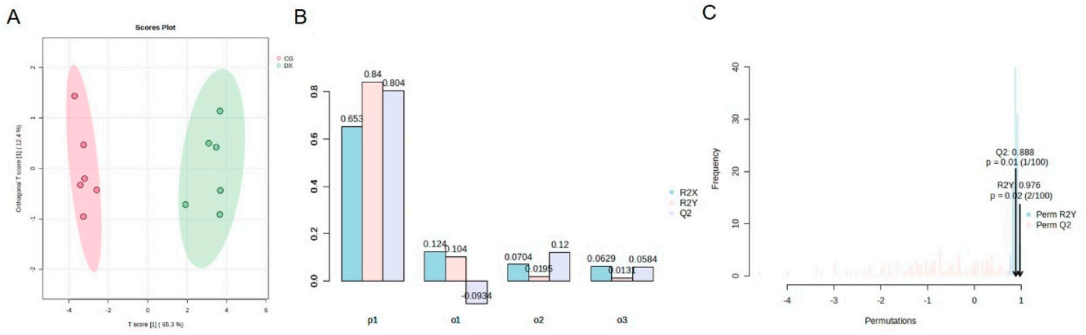


**Figure S3** OPLS-DA model analysis based on 18 metabolites. (A): the OPLS-DA model based on multi-element fingerprint; (B): the cross validation variance of (A); (C): shows the results of 100 permutation tests of (A); CG represents rice obtained under conventional cultivation mode; DX represents rice obtained under rice crab co cultivation mode.
